# Visual Familiarity Induced 5-Hz Oscillations and Improved Orientation and Direction Selectivities in V1

**DOI:** 10.1523/JNEUROSCI.1337-20.2021

**Published:** 2021-03-24

**Authors:** Mang Gao, Sukbin Lim, Alexander A. Chubykin

**Affiliations:** ^1^Department of Biological Sciences, Purdue Institute for Integrative Neuroscience, Purdue Autism Research Center, Purdue University, West Lafayette, Indiana 47907; ^2^Neural Science, New York University Shanghai, Shanghai, 200122, China; ^3^New York University-East China Normal University Institute of Brain and Cognitive Science, New York University Shanghai, Shanghai, 200062, China

**Keywords:** awake patch clamp, experience, familiarity, oscillations, selectivity, visual cortex

## Abstract

Neural oscillations play critical roles in information processing, communication between brain areas, learning, and memory. We have recently discovered that familiar visual stimuli can robustly induce 5-Hz oscillations in the primary visual cortex (V1) of awake mice after the visual experience. To gain more mechanistic insight into this phenomenon, we used *in vivo* patch-clamp recordings to monitor the subthreshold activity of individual neurons during these oscillations. We analyzed the visual tuning properties of V1 neurons in naive and experienced mice to assess the effect of visual experience on the orientation and direction selectivity. Using optogenetic stimulation through the patch pipette *in vivo*, we measured the synaptic strength of specific intracortical and thalamocortical projections *in vivo* in the visual cortex before and after the visual experience. We found 5-Hz oscillations in membrane potential (V_m_) and firing rates evoked in single neurons in response to the familiar stimulus, consistent with previous studies. Following the visual experience, the average firing rates of visual responses were reduced while the orientation and direction selectivities were increased. Light-evoked EPSCs were significantly increased for layer 5 (L5) projections to other layers of V1 after the visual experience, while the thalamocortical synaptic strength was decreased. In addition, we developed a computational model that could reproduce 5-Hz oscillations with enhanced neuronal selectivity following synaptic plasticity within the recurrent network and decreased feedforward input.

**SIGNIFICANCE STATEMENT** Neural oscillations at around 5 Hz are involved in visual working memory and temporal expectations in primary visual cortex (V1). However, how the oscillations modulate the visual response properties of neurons in V1 and their underlying mechanism is poorly understood. Here, we show that these oscillations may alter the orientation and direction selectivity of the layer 2/3 (L2/3) neurons and correlate with the synaptic plasticity within V1. Our computational recurrent network model reproduces all these observations and provides a mechanistic framework for studying the role of 5-Hz oscillations in visual familiarity.

## Abstract (precis)

Neural oscillations play critical roles in information processing, communication between brain areas, learning, and memory. We have recently discovered that familiar visual stimuli can robustly induce 5-Hz oscillations in the primary visual cortex (V1) of awake mice after the visual experience. To gain more mechanistic insight into this phenomenon, we used *in vivo* patch-clamp recordings to monitor the subthreshold activity of individual neurons during these oscillations.

## Introduction

Neural oscillations at around 5 Hz have been observed in the neocortex in both rodents and primates. These oscillations are involved in a variety of cognitive functions. In the auditory cortex, they provide temporal windows for processing syllables (Luo and Poeppel, 2007; Peelle et al., 2013; Fuentemilla et al., 2014). These oscillations coordinate the precuneus and medial prefrontal cortex with the medial temporal lobe during autobiographical events retrieval (Fuentemilla et al., 2014). In the visual cortex, oscillations at around 5 Hz have been observed during learning in awake animals. In primates, 4- to 8-Hz phase-locked single-unit activity was present during visual working memory tasks within V4 (Lee et al., 2005). Five-Hertz phase-locked γ synchronization has been shown to correlate with short-term memory capacity (Sauseng et al., 2009). Three- to 9-Hz phase synchrony of V4 and the lateral prefrontal cortex was shown to be predictive of the behavioral performance (Liebe et al., 2012). In rodents, 4- to 8-Hz oscillations in the primary visual cortex (V1) were shown to predict the timing of a visually cued reward (Zold and Hussain Shuler, 2015; Levy et al., 2017). In contrast to reward prediction, 3- to 5-Hz oscillations have also been reported to reduce the visual responses recorded in mouse V1 (Einstein et al., 2017). We recently discovered that familiar visual stimuli could robustly induce 4- to 8-Hz oscillations in V1 of mice that have undergone the visual experience (Kissinger et al., 2018). These familiarity-triggered oscillations were not driven by the temporal modulation of the stimuli and could be induced by a static stimulus. These studies suggest that oscillations in the visual cortex may play vital roles in visual learning and information processing. However, the exact physiological function of such oscillations in V1 and their underlying mechanisms remain unclear.

To dissect the role of 5-Hz oscillations in experience-dependent plasticity in V1 and gain more mechanistic insight into how the oscillations modify visual responses at the cellular level, we performed *in vivo* patch-clamp recordings in naive and experienced mice. We discovered that oscillations of the membrane potential (V_m_) at around 5 Hz, and bursts of action potentials (APs), were evoked in single neurons in response to the familiar stimulus after the visual experience along with a decreased stimulus-evoked firing. To test whether the selectivity of V1 neurons was modulated, we recorded their responses to 12 directions of drifting sinusoidal gratings and measured both orientation selectivity (OS) and direction selectivity (DS). Although the firing rates of visual responses to all directions and orientations of sinusoidal drifting gratings were reduced, the OS index (OSI) and DS index (DSI) were increased. To assess the synaptic strength changes resulting from the visual experience, we used optogenetic measurements through the patch pipette *in vivo* to measure the synaptic strength of the thalamocortical and intracortical projections in naive mice and experienced mice. In the experienced mice, light-evoked EPSCs were significantly increased for the intracortical projections from layer 5 (L5) to other layers of V1, while the strength of the thalamocortical synapses was decreased. Finally, we developed a computational recurrent network model describing how synaptic plasticity observed experimentally can account for the effects of visual experience on stimulus selectivity and dynamic properties.

## Materials and Methods

### 

#### Animals

All animal use was approved by the Purdue University animal care and use committee. All mice were housed in a 12/12 h light/dark cycle with full access to food and water. The mice used were: C57BL/6 (The Jackson Laboratory), Thy1-ChR2-YFP (B6.Cg-Tg(Thy1-COP4/EYFP)18Gfng/J, The Jackson Laboratory), Vglut2-ChR2-YFP(C57BL/6-Tg(Slc17a6-COP4*H134R/EYFP)2Oki/J, The Jackson Laboratory). All mice used in experiments were at postnatal days 58–62. A total of 76 mice were used, including 36 male and 40 female mice.

#### Surgical procedure

At postnatal day 53, age matched mice were selected for head-plate implantation. Anesthesia was induced by 5% isoflurane in air and maintained at 1.5% isoflurane during surgery. Animals were head-fixed in a motorized stereotaxic apparatus (Neurostar). The surgery area was shaved and sterilized with ethanol. Ophthalmic ointment was applied to the eyes. A custom-designed head-plate was fixed to the skull with adhesive cement (C&B Metabond). The recording site (from λ: anteroposterior 0.3 mm, mediolateral 3.0 mm) was marked and covered by Kwik-Cast Silicone Elastomer. Before recording, a 0.04 mm^2^ square craniotomy window was made and filled with artificial CSF (ACSF; 1.25 mm NaH_2_PO_4_, 26 mm NaHCO_3_, 10 mm dextrose, 124 mm NaCl, 1 mm CaCl_2_, 0.8 mm MgCL_2_, and 3.5 mm KCl). For recordings in awake mice, mice recovered in the air for 30 min after isoflurane removal. For recordings in anesthetized mice, mice were kept anesthetized with intraperitoneally injected ketamine (100 mg/kg)/xylazine (16 mg/kg).

#### Perfusion and histology

Mice were anesthetized with intraperitoneally injected ketamine (100 mg/kg)/xylazine (16 mg/kg). They were transcardially perfused with 1× Phosphate-buffered saline (PBS) and followed by 4% paraformaldehyde (PFA). The brain was extracted and placed in 4% PFA for 20 h before slicing. The fixed brain was sliced into 50-μm coronal sections with a vibratome (TPI 1000 Plus). The images of brain sections were acquired with a LSM 710 confocal microscope and a 20×/1.0 NA lens (Zeiss, Plan-Apochromat) in tile scan mode.

#### Electrophysiology

*In vivo* patch-clamp recordings were performed in awake head-fixed mice. Patch pipettes (filamented borosilicate glass (BF150-86-10, Sutter Instrument) of 4–7 MΩ were pulled (P-97, Suter Instrument) and filled with internal solution (130 mm potassium D-gluconate, 5 mm KCl, 2 mm MgCl_2_, 0.3 mm NaGTP, 10 mm HEPES, and 0.6 mm EGTA). Electrophysiological recordings were acquired with an amplifier (Multiclamp 700B, Molecular Devices) and a digitizer (Digitata, 1550, Molecular Devices). Acquired data were collected using Clampex (Molecular Devices) with a 10,000-Hz low-pass filter. Blind patch-clamp recordings were performed with the assistance of an open-source software, Autopatcher (Wu et al., 2016; Wu and Chubykin, 2017). Cells with access resistance >60 MΩ were excluded. Membrane capacitance was corrected and compensated once a steady patch was formed.

#### Optogenetic stimulation

A blue laser (Opto Engine, 100 mW, 473 nm) was used as the light source for optogenetic stimulation. For all optogenetic experiments, Optopatcher (AM System; Katz et al., 2013) was used to replace the standard pipette holder. The light was delivered with a custom cut and polished optical fiber (Thorlabs, 0.39 NA TECS hard-clad, multimode, step-index fibers, FT200EMT). The optical fiber was inserted into a glass pipette within 1 mm from the pipette tip and was located coaxially to the pipette. A 2-mW laser power (measured at the internal solution filled pipette tip) was used for all experiments. The laser output power was calibrated before and after experiments to make sure the same power was used for all recordings.

#### Visual stimulation

Visual stimulation was generated and controlled by custom python scripts using the open-source PsychoPy package. Visual stimulation was presented on a γ calibrated LCD monitor. The gray background that was presented before and after the stimuli had the same luminance as the stimuli. A sinusoidal grating stimulus (200 ms, 0.04 cycle/°, 2 Hz) was used for training and recording. For orientation and direction tuning recordings, the grating stimulus was rotated in 30° steps to create the stimuli with 12 drifting directions. All stimuli were presented at 100% contrast.

#### Data analysis

For the analysis of V_m_, APs were removed from raw traces with a 12.5-ms median filter. The V_m_ power spectra over frequencies were computed with Fast Fourier transform. The magnitudes of frequency bands were computed using bandpass filters on the spike-removed traces. The time-frequency analysis was computed with complex wavelet convolution (Cohen, 2014). A total of 40 frequencies across a logarithmic range from 2 to 80 Hz and 3 to 10 cycles of the wavelet were used. The coherence of frequency bands was computed with the same complex wavelet convolution method.

To detect 5-Hz V_m_ oscillation, we took the traces from the stimulus onset to 0.5 s after the onset as visual responses, and the traces from 4.5 to 5 s after the stimulus onset as the baseline. The trials that the visual response had >10 times 4- to 7-Hz power of the averaged baseline was determined as oscillation trials. We also manually inspected all trials to ensure the detection was correct (Einstein et al., 2017).

To detect APs, two smoothed traces were generated by convolving the raw trace with a Hanning window of 0.5 or 500 ms, respectively. The coarsely smoothed trace was subtracted from the finely smoothed trace to remove the V_m_ fluctuation for spike detection. The times of the peaks exceeding 15 mV were detected as the time of APs. Peristimulus time histograms (PSTHs) were computed from AP times with a 10-ms bin size. The PSTH was convolved with a 200-ms Gaussian window with 40-ms σ and normalized for each cell to calculate the normalized firing rate. The APs fired during 50–250 ms after the stimulus onset were considered visual response for 200-ms stimulus. The APs fired from 50 to 550 ms after the stimulus onset were considered visual response for 500-ms stimulus. To compute the OSI and DSI, we randomly split the trials into two halves. The preferred direction was computed from the one-half of the trials. The preferred direction was used together with the other half of the trials to compute the OSI and DSI. The OSI and DSI were computed as follows:

$$OSI=\frac{R_{pref}-R_{orth}}{R_{pref}+R_{orth}},$$

$$DSI=\frac{R_{pref}-R_{opp}}{R_{pref}+R_{opp}},$$

where $R_{pref}$ is the mean firing rate of the response to the preferred direction, $R_{orth}$ is the mean firing rate of the response to the orthogonal direction, the $R_{opp}$ is the mean firing rate of the response to the opposite direction. This procedure was repeated 2000 times to generate the averaged OSI and DSI. The repetitions that generated a negative number were disregarded, as the preferred direction was unreliable in such a case.

To extract the phase angle of APs, a bandpass filter was applied to the spike-removed traces to extract the 4- to 7-Hz V_m_ activities. The phase angles of every time point were computed with a Hilbert transform. The phases of APs within the visual response window were extracted.

To extract the light-evoked EPSC, a 0.2-s baseline before the onset of the stimulus was selected for each trial. The EPSP peaks were detected from 0 to 40 ms after the stimulus onset. The difference from the baseline to the EPSC peak was defined as the EPSC amplitude. The EPSC latency was defined as the time interval between center the TTL signal of the stimulus and the time of the EPSC reached 5% of the amplitude (Boudkkazi et al., 2007). We only used the cells that had <3.5-ms average EPSC latency.

All data analysis was performed in Python. The source code is available from the corresponding author on reasonable request.

#### Network simulation

In the model, the activity of, and synaptic interactions between, the neurons are parametrized by their preferred direction φ which is uniformly distributed along a ring between –π and π. To account for enhanced low-frequency oscillation with visual learning, slow negative feedback such as firing rate adaptation was included as in the previous work (Lim, 2019). The dynamics of network activity is described by the following equations

$$\tau_{r}\frac{dr(\phi ,t)}{dt}=-r(\phi ,t)+f\left(\int_{-\pi }^{\pi }J(\phi ,\phi^{\prime })r(\phi^{\prime },t)d\phi^{\prime }+i(\phi ,t)-ka(\phi ,t)\right)\tau_{a}\frac{da(\phi ,t)}{dt}=-a(\phi ,t)+r(\phi ,t),$$

where r(ϕ,t) and a(ϕ,t) represent the mean firing rate and adaptation variable of populations with the preferred feature ϕ. r(ϕ,t) approaches f(x(ϕ,t)) with intrinsic time constant $\tau_{r}$, where *f(x)* is the steady-state neuronal response to input current *x*. Here, we considered a threshold nonlinear function, that is, *f(x) = x* when *x* ≥ 0, and otherwise 0.

The input x(ϕ,t)to a population with the preferred feature ϕ is a sum of the recurrent synaptic currents $J(\phi ,\phi^{\prime })r(\phi^{\prime },t)$ with the preferred feature $\phi^{\prime }$, the feedforward current i(ϕ,t) minus the adaptation current ka(ϕ,t). We considered a linear mechanism for adaptation such that ka(ϕ,t)is a low-pass filtered firing rate r(ϕ,t)with time constant $\tau_{a}$ and strength *k*. In the recurrent inputs, $J(\phi ,\phi^{\prime })$ represents the synaptic connectivity strength, which depends only on the distance between ϕ and $\phi^{\prime }$. Thus, it can be written as $J(\phi -\phi^{\prime })$and we assumed that $J(\phi -\phi^{\prime })$is the sum of Gaussian functions as $J_{E}\exp\left[-{\left(\phi -\phi^{\prime }\right)}^{2}/\sigma_{E}{}^{2}\right]-J_{I}\exp\left[-{\left(\phi -\phi^{\prime }\right)}^{2}/\sigma_{I}{}^{2}\right]$ where the subscripts *E* and *I* represent the recurrent excitation and inhibition. The external current i(ϕ,t) is modeled as a product of a spatial component $i_{s}(\phi )$ and a temporal component $i_{t}(t)$ so that $i(\phi ,t)=i_{s}(\phi )i_{t}(t)$. The temporal component $i_{t}(t)$ is a pulse of duration $t_{stim}$ that is exponentially filtered with time constant $\tau_{ext}$. The spatial component $i_{s}(\phi )$is the sum of two Gaussian functions with the same width and centered at the stimulus direction $\phi_{0}$ and its opposite direction with two modulation factors $\gamma_{0}$ and $\gamma_{1}$ as $i(\phi )=\gamma_{0}\left(\exp\left[-{\left(\phi -\phi_{0}\right)}^{2}/\sigma_{stim}{}^{2}\right]+\gamma_{1}\exp\left[-{\left(\phi -\phi_{0}+\pi \right)}^{2}/\sigma_{stim}{}^{2}\right]\right)$.

The parameters used in the simulation are following – the time constants and parameters for the adaptation and the feedforward inputs except the overall modulation factor $\gamma_{0}$ did not change with learning, given as $\tau_{r}$ = 5 ms, $\tau_{a}$ = 150 ms, $\tau_{ext}$ = 50 ms, $t_{stim}$ = 500 ms, k = 1, $\sigma_{stim}$ = π/10 and $\gamma_{1}$ = 0.2. Learning induced changes in the recurrent connections and overall feedforward strengths such that before learning, $\gamma_{0}$ = 1, $J_{E}$ = 0.15, $\sigma_{E}$ = ∞, $J_{I}$ = 0, and after learning, $\gamma_{0}$ = 0.5, $J_{E}$ = 3, $\sigma_{E}$ = π/6, $J_{I}$ = 1, $\sigma_{I}$ = ∞. Here, σ= ∞ for Gaussian functions represents a constant profile and the parameters for adaptation currents were chosen to generate the 5-Hz oscillation after learning. The simulation was run with a fourth-order explicit Runge–Kutta method in MATLAB, and the source code will be available to anyone interested.

#### Statistical analysis

For recording with only single direction stimulus, we used two-sided Mann–Whitney *U* tests for comparison on band magnitude, ΔV_m_, firing rate, and oscillation probability. For 12 direction tuning data, we performed mixed-design two-way ANOVA with directions as a within-subject factor and visual experience as a between-subject factor. Mixed-design two-way ANOVA was performed on firing rate, band magnitude, band coherence, and the AP phase angles. Mauchly's sphericity test was used to determine whether a Greenhouse–Geisser correction was needed. We used two-sided Mann–Whitney *U* tests for pair-wise comparison after ANOVA. Benjamini–Hochberg FDR correction was used whenever a correction on the p-value was required. Two-sided Mann–Whitney *U* tests were also used to compare OSI, DSI, and the light-evoked EPSP amplitudes of naive and experienced mice. All statistical analysis was performed using Pingouin, an open-source statistic Python package.

## Results

### Visual familiarity induced oscillations reduced visual responses in V1

**Figure 1.**
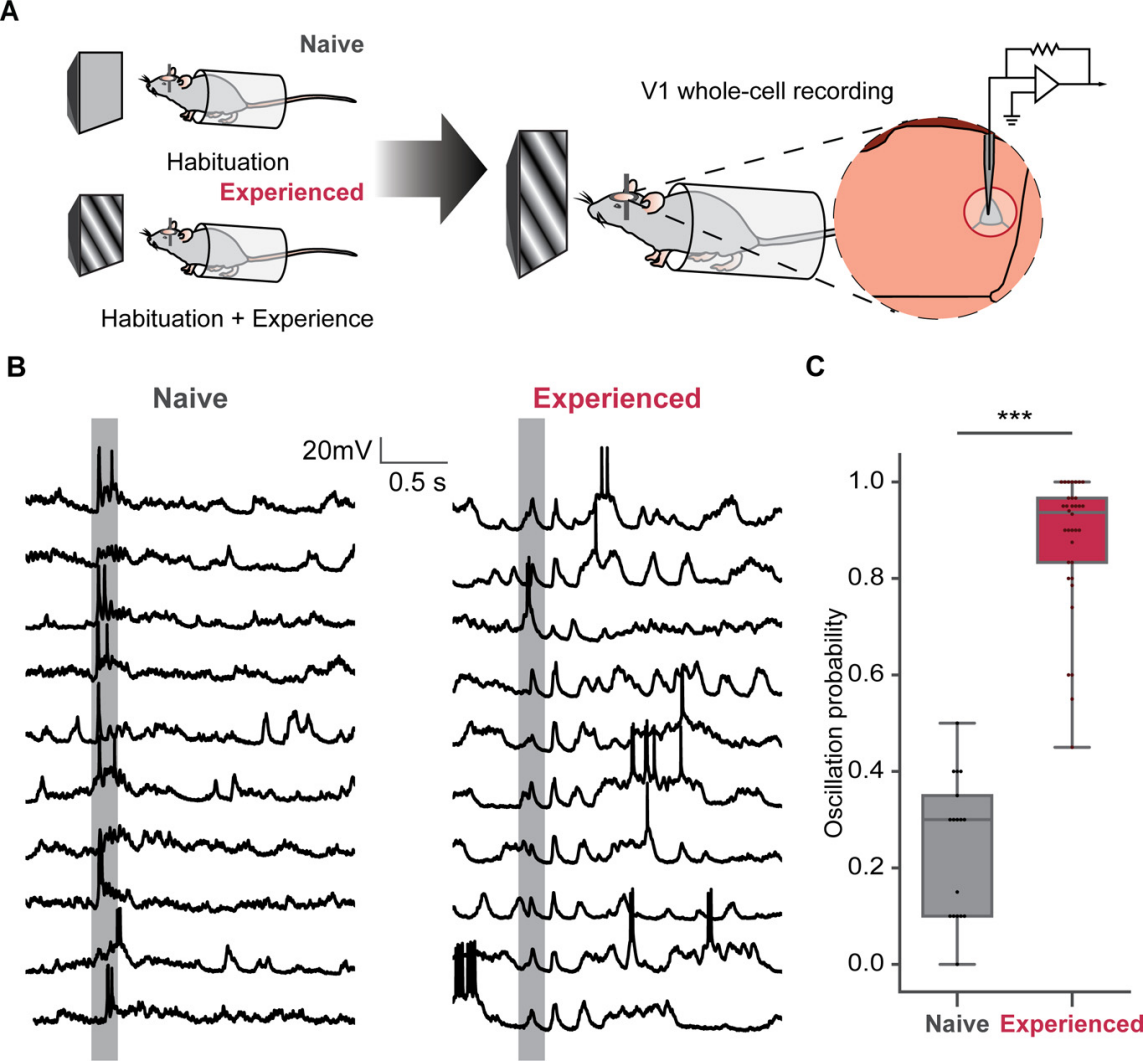
Five-Hertz oscillations evoked by familiar visual stimulus. ***A***, Experimental setup and passive visual experience paradigm. After 2 d of habituation, mice were either presented with the sinusoidal grating stimulus or habituated for four more days. *In vivo* whole-cell recordings were performed on awake mice. ***B***, Representative traces of cells responding to the stimulus in naive and experienced mice. Gray rectangles represent the visual stimuli duration. ***C***, Box plot and swarm plot of oscillation probabilities of cells recorded in naive and experienced mice; ***p < 0.001.

**Figure 2.**
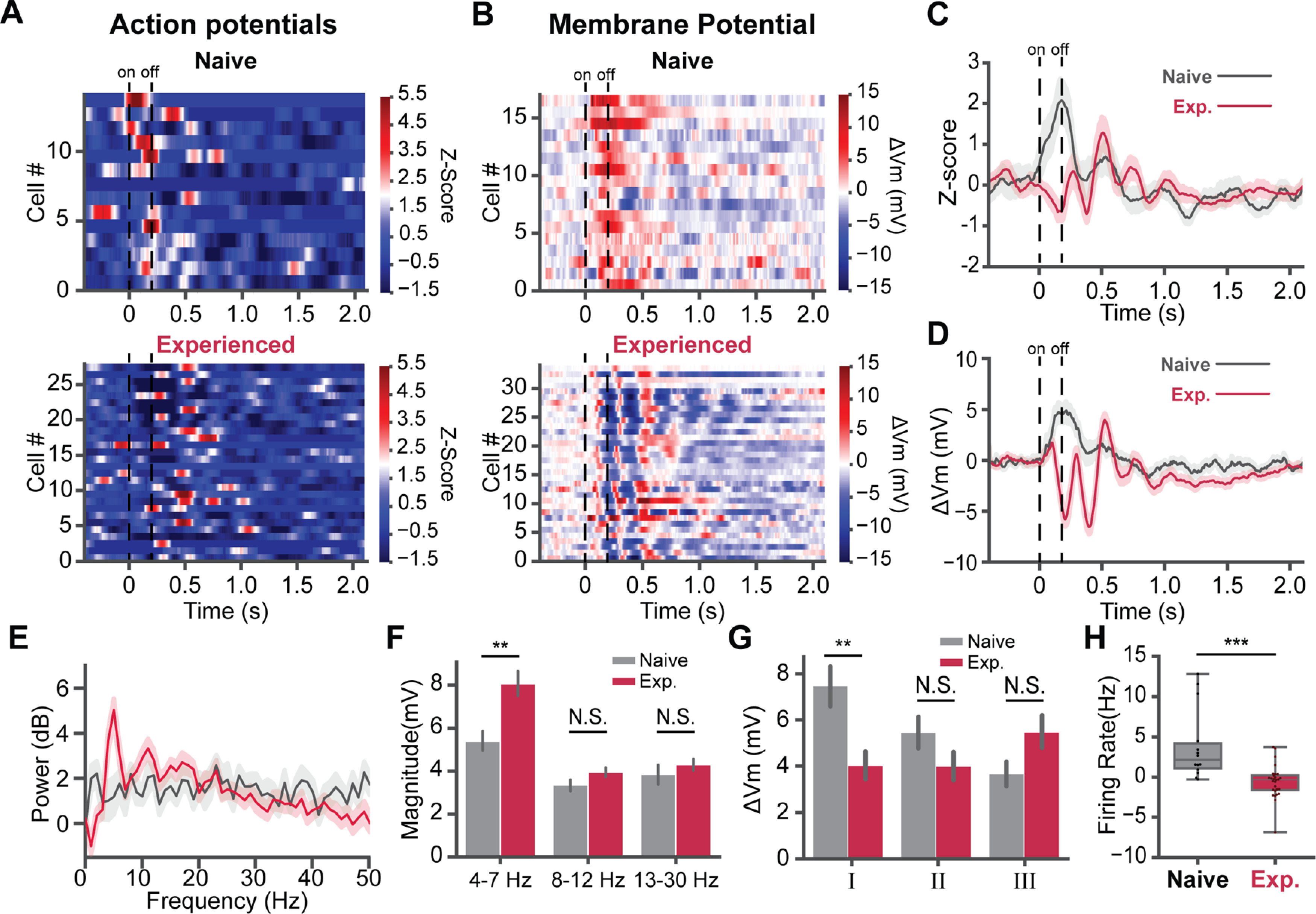
Five-Hertz oscillations correlate with the decreased firing rate of neural responses. ***A***, Z-scored firing rate of cells in naive (top, 14 cells) and experienced mice (bottom, 28 cells). Dashed lines indicate the stimulus onset/offset. ***B***, Baseline-subtracted V_m_ (Δ V_m_) of responses in naive (top, 17 cells) and experienced mice (bottom, 34 cells). ***C***, Average z-scored firing rate of neurons in naive (gray, 14 cells) and experienced (crimson, 28 cells) mice. The shaded area represents the SEM. ***D***, The averaged ΔV_m_ of cells in naive (gray, 17 cells) and experienced (crimson, 34 cells) mice. ***E***, Cell averaged power spectra of V_m_ subthreshold responses for naive (gray, 17 cells) and experienced mice (crimson, 34 cells). ***F***, V_m_ powers in several frequency bands. The average magnitude of 4- to 7-, 8- to 12-, and 13- to 30-Hz bands were computed (naive, 17 cells; experienced, 34 cells). ***G***, Peaks of baseline subtracted V_m_ (ΔV_m_) from the first (I), second (II), and third (III) time windows. Each window corresponds to a cycle of the 5-Hz oscillation in experienced mice. ***H***, The average firing rate of cells in naive (14 cells) and experienced mice (28 cells). Exp., experienced. The baseline firing rate was subtracted from each cell; *p < 0.05, **p < 0.01, ***p < 0.001; N.S., not significant. Error bars indicate mean ± SEM.

To dissect the mechanism of visual familiarity evoked 5-Hz oscillations, we performed whole-cell patch-clamp recordings in L2/3 of V1 in awake mice. Age-matched mice were divided into two groups. After 2 d of habituation, one group (naive) was habituated with gray screen for four more days; the other group (experienced) was familiarized with a sinusoidal grating stimulus (200 ms, 0.04 cycle/°, 2 Hz, 200 repeats/d, 4 d) via passive viewing (Fig. 1*A*). The visual responses of L2/3 neurons to the same grating stimulus were recorded from both groups. The stimulus was novel to the naive mice that only habituated with the gray screen but was familiar to the experienced mice that passively viewed it for 4 d. The mice were awake during the recordings. We successfully recorded cells from nine naive and 16 experienced mice. A total of 17 cells recorded from the naive mice and 34 cells recorded from the experienced mice had subthreshold visually evoked responses on V_m_. Among these cells, three cells in naive mice and six cells in experienced mice had no superthreshold responses. They were discarded in the superthreshold analysis. While the V_m_ oscillations can also be found in some trials in the naive mice (Fig. 1*C*), the ratio of oscillation trials greatly increased in the experienced mice (Mann–Whitney *U* test, *U* = 1.0, *p* = 4.06 × 10^−9^). In some cells from experienced mice, the 5-Hz oscillations occurred in all trials following the visual stimulus onset (Fig. 1*B*,*C*). In naive mice, neurons responded to the novel visual stimulus with a strong peak of firing, followed by a smaller second peak (Fig. 2*C*). Consistent with our previous work (Kissinger et al., 2018), the visual stimulus-evoked oscillatory APs responses extended beyond the stimulus in experienced mice (Fig. 2*A*, lower, *C*). Most cells preferred to fire at the second and the third cycles of the oscillation. Meanwhile, firing during the first cycle of oscillation was significantly reduced. We computed the baseline- subtracted firing rate of all cells, using 0.05–0.25 s after the stimulus onset as the visual response time window and 2.5–4.7 s after the onset as the baseline. The decreased firing rate of the visual response in experienced (*n* = 14 cells) mice was significant compared with naive (*n* = 28 cells) mice (*U* = 347.0, *p* = 5.9 × 10^−5^; Fig. 2*H*).

In addition to the APs' oscillatory activity, the subthreshold V_m_ oscillations were also prominent in the responses of experienced mice (Fig. 2*B*, lower). The visually evoked oscillations in the V_m_ lasted for three to four cycles in most neurons following the stimulus (Fig. 2*B*, lower, *D*) in experienced mice. In naive mice, V_m_ had no oscillations, but rather a broad depolarization following the visual stimulus (Fig. 2*B*, upper). The power spectra of V_m_ (Fig. 2*E*) showed a significant increase in power around 5 Hz. The magnitude of 4- to 7-Hz band V_m_ significantly increased in experienced mice compared with naive mice (naive, *n* = 17 cells; experienced, *n* = 34 cells; Mann–Whitney *U* test, *U* = 148.0, *p* = 0.0050; Fig. 2*F*). No significant changes happened in the 8- to 12-Hz (*U* = 201.0, *p* = 0.080) and 13- to 30-Hz (*U* = 224.0, *p* = 0.20) oscillations. We took the times of the first three cycles of 5-Hz V_m_ oscillation in experienced mice, and defined three time windows (I, 0.05–0.25 s; II, 0.25–0.45 s; III, 0.45–0.65 s after stimulus) to quantify the V_m_ peaks for each cycle. The average V_m_ of 2.5–4.7 s after the stimulus onset was subtracted from the V_m_ peaks. The baseline subtracted peak V_m_ (ΔV_m_) was compared with the averaged maximum ΔV_m_ within the same time windows in naive mice (Fig. 2*G*). The membrane oscillation strongly suppressed V_m_ during the visual stimulus. The peak ΔV_m_ of the first cycle in experienced mice was lower than the peak ΔV_m_ of the same time window in naive mice (*U* = 450.0, *p* = 0.0013). Meanwhile, there was no significant difference in ΔV_m_ at the time window of the second (*U* = 368.0, *p* = 0.12) and third cycle (*U* = 212.0, *p* = 0.13) between the two groups (Fig. 2*G*).

### Visual experience improves the orientation and direction selectivities of V1 neurons

**Figure 3.**
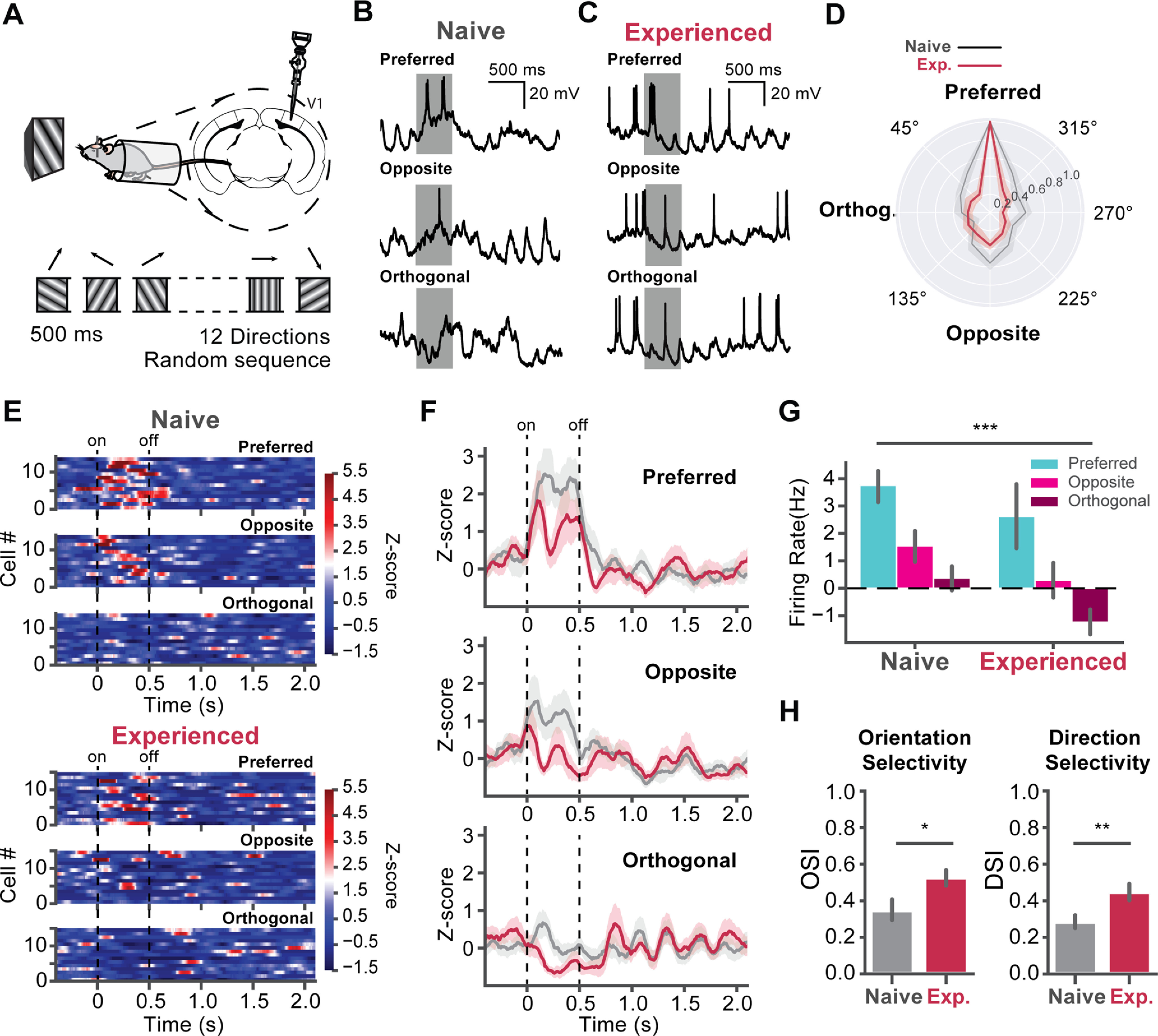
Visual experience improves the direction selectivity in V1. ***A***, Experimental design for direction tuning in V1. The naive and experienced mice were recorded with 500-ms sinusoidal grating stimuli with 12 directions in pseudorandom sequences. ***B***, Example traces of a cell responding to the preferred, opposite, and orthogonal visual stimuli. ***C***, Same as ***B***, but for a cell in an experienced mouse. ***D***, Normalized responses of cells in naive (gray, 14 cells) and experienced (crimson, 15 cells) mice for 12 directions. The shaded areas represent SEM of the responses. ***E***, Heatmaps of z-scored firing rates of cells in naive and cells in experienced mice. The color bar shows the *z*-score scale. ***F***, The time courses of averaged z-scored firing rates of V1 cells in naive and experienced mice. ***G***, The average firing rate during visual stimulus of the naive and experienced mice. The baseline firing rate was subtracted. The firing rate significantly decreased in the experienced compared with naive mice. ***H***, left, Bar plots of the OSI. The OSI of cells in the naive (gray) and experienced mice (crimson). Right, Bar plot of DSI; *p < 0.05, **p < 0.01, ***p < 0.001; N.S., not significant. Error bars indicate mean ± SEM.

**Figure 4.**
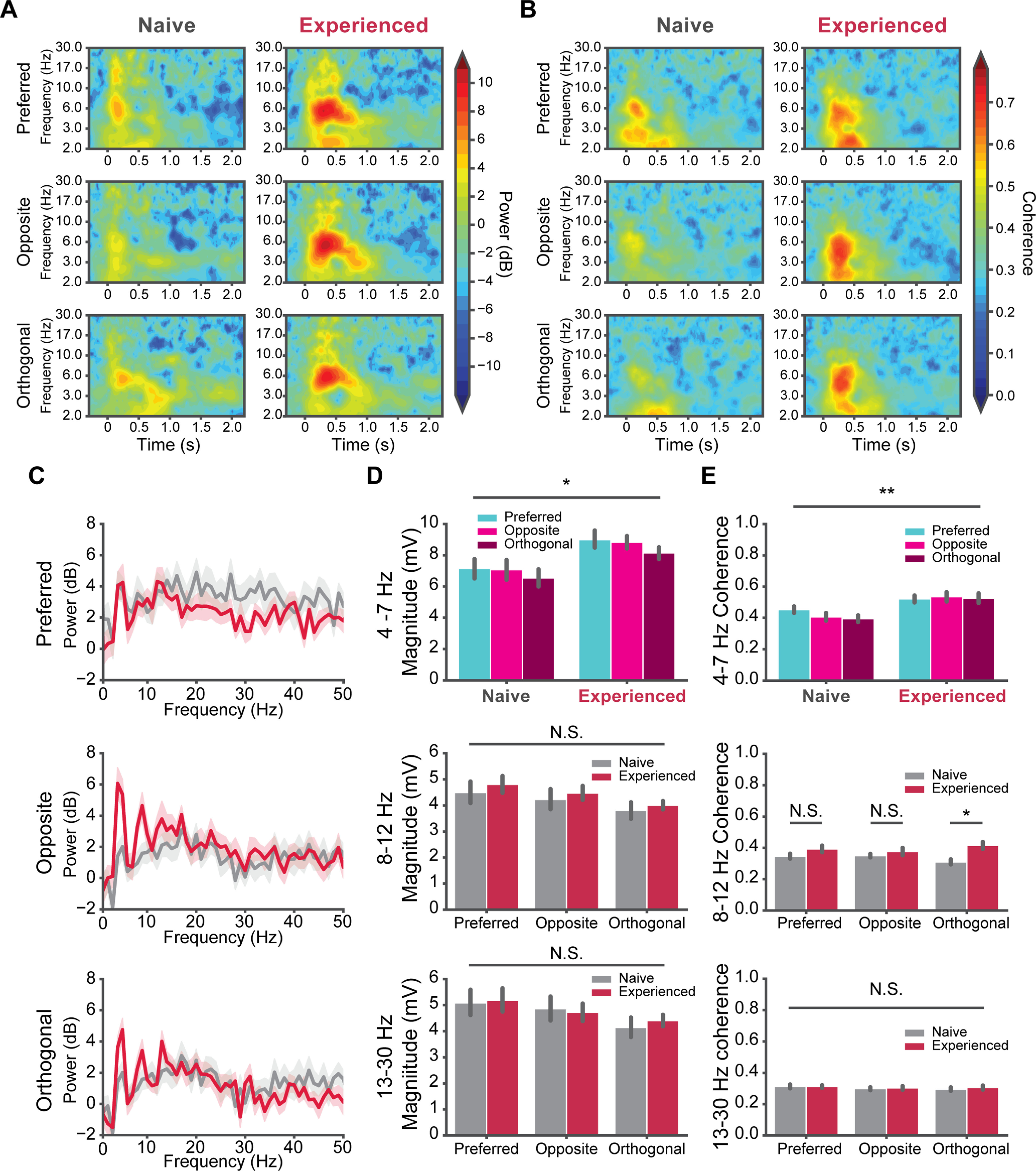
V_m_ oscillations during V1 direction tuning. ***A***, Time-frequency spectrograms of the V_m_ responses to visual stimuli. The V_m_ of each cell responding to preferred, opposite, and orthogonal direction were converted to the time and frequency domain by wavelet transform. The heat in each spectrogram represents the baseline normalized power in dB. Left column, Naive mice. Right, Experienced mice. ***B***, The ITPC of V_m_ responses to the preferred, opposite, and orthogonal directions. Left column, Naive mice. Right column, Experienced mice. The heat represents the ITPC. ***C***, Average power spectra of V_m_ responses to the preferred, opposite, and orthogonal direction naive (gray) and experienced mice (red). ***D***, top, The 4- to 7-Hz magnitude of cells in naive and experienced mice for the preferred, opposite and orthogonal directions. Middle, Same as the top, but for the α band (8–12 Hz). Bottom, Same as top but for the β band (13–30 Hz). ***E***, The averaged ITPC of V_m_ during the visual stimuli in naive and experienced mice. Top, 4- to 7-Hz ITPC; middle, α band ITPC; bottom, β band ITPC; *p < 0.05, **p < 0.01, ***p < 0.001; N.S., not significant. Error bars indicate mean ± SEM.

Our results showed that visual experience led to the emergence of the 5-Hz oscillations and a decreased population-averaged firing rate during the visual stimulus. However, the reduced firing rate could result in a decreased feature selectivity because of the reduced visual responsiveness; or it could result in an increased feature selectivity by increasing the preferred/non-preferred response ratio. In previous studies, enhanced selectivity of V1 neurons has been found in conjunction with perceptual learning and reward training with visual cues (Schoups et al., 2001; Poort et al., 2015; Khan et al., 2018). To determine whether the feature selectivity of L2/3 neurons was enhanced after the visual experience, we next recorded responses to 12 directions of drifting sinusoidal gratings in awake mice. Age-matched mice were separated into two groups, habituated and familiarized to the specific visual stimulus as described in the previous experiment. Sinusoidal grating stimuli of a 500-ms duration with the same spatial (0.04 cycle/°) and temporal frequency (2 Hz) of 12 drifting directions (30° between each direction) were used for orientation and direction tuning recordings (Fig. 3*A*). Stimuli of 12 different directions were presented in a pseudorandom sequence with 3.5 s intervals. We were able to record 14 cells from 11 naive mice and 15 cells from 12 experienced mice. Surprisingly, the V_m_ oscillations occurred in responses to visual stimuli of all directions in the experienced mice (Figs. 3*C*, 4*D*), not only to the familiar direction used in passive viewing (60° to horizontal). To quantify the superthreshold visual response, 0.05–0.55 s after visual stimuli onset was used as the visual response time window for APs. The 2.5–3.5 s after the onset was used as the baseline and subtracted from each trial. The baseline-subtracted firing rates of preferred, opposite, and orthogonal directions in experienced mice were significantly lower compared with naive mice (*U* = 1354.0, *p* = 0.00052), No interaction between the direction and visual experience was found (naive, *n* = 14 cells; experienced, *n* = 15 cells; two-way ANOVA, *F* = 0.091, *p* = 91.33; Fig. 3*F*,*G*). To determine whether the DS and OS of V1 neurons were changed, we computed the OSI and DSI of recorded neurons (Fig. 3*H*). OSI increased in the experienced mice that were familiarized with the stimulus (*U* = 49.0, *p* = 0.015). The DSI also increased in the experienced mice (*U* = 42.0, *p* = 0.0064).

We then performed time-frequency analysis on V_m_ (Fig. 4*A*), and extracted the powers of the 4- to 7-, 8- to 12-, and 13- to 30-Hz bands for the preferred, opposite, and orthogonal directions. We also computed the averaged power spectrum of V_m_ in the visual response time window (Fig. 4*C*) and the band magnitudes by bandpass filtering V_m_. For all three directions, the 4- to 7-Hz amplitude significantly increased in the experienced mice compared with naive mice (*U* = 54.0, *p* = 0.028; Fig. 4*D*, top). No interaction between the directions and visual experience was found (two-way ANOVA, *F* = 0.0813, *p* = 0.92). The visual experience did not affect the 8- to 12-Hz band (*F* = 0.336, *p* = 0.567) and 13- to 30-Hz band (*F* = 0.0192, *p* = 0.89). We also computed the intertrial phase coherence (ITPC) of the V_m_ visual responses (Fig. 4*B*). The 4- to 7-Hz ITPC increased in the experienced mice (*U* = 31.0, *p* = 0.0013; Fig. 4*E*, top), while no interaction between directions and visual experience was found (*F* = 1.464, *p* = 0.24). The cell averaged ITPC reached ∼0.7 in experienced mice. Such strong coherence suggests the oscillation was strongly time-locked to the visual stimuli. The coherence of 8- to 12-Hz band also increased in the experienced mice when the orthogonal stimulus was present (two-way ANOVA, *F* = 2.06, *p* = 0.012, pair-wise *U* test, *U* = 38.0, *p* = 0.011; Fig. 4*E*, middle), and was unchanged for preferred (*U* = 62.0, *p* = 0.095) and opposite direction (*U* = 89.0, *p* = 0.50). The ITPC of 13- to 30-Hz band remained low and unchanged in naive and experienced mice (*F* = 0.195, *p* = 0.66; Fig. 4*E*, bottom).

**Figure 5.**
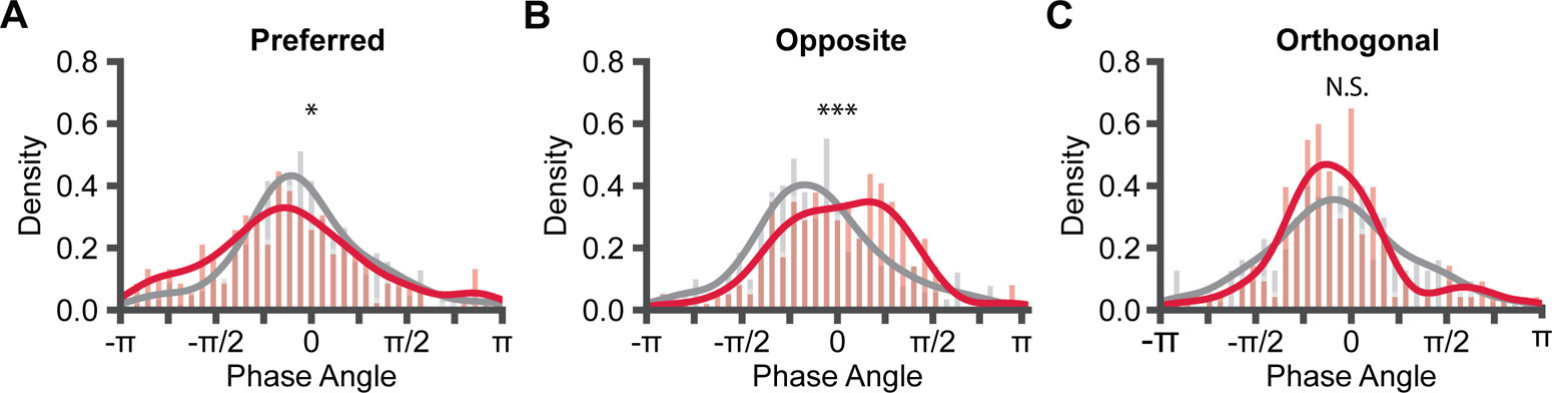
AP phase distributions during V_m_ oscillations. ***A***, Density histograms of APs 4- to 7-Hz phase angle distribution; 0 phase angle represents the peak of 4- to 7-Hz V_m_. Negative phase angles represent the rising phase, and positive phase angles represent the falling phase. ***B***, ***C***, Same as ***A***, but for the opposite and orthogonal directions; *p < 0.05, ***p < 0.001; N.S., not significant.

To quantify the phase-locking activity of APs, we filtered the V_m_ traces to extract the 4- to 7-Hz V_m_ and transferred the filtered signals to analytical signals using Hilbert transform. The phase angle of APs was extracted. The APs shifted toward the rising phase of the V_m_ oscillation for the preferred direction in the experienced mice (two-way ANOVA, *F* = 8.078, *p* = 0.00032; pair-wise *U* test, *U* = 79,427.0, *p* = 0.027; Fig. 5*A*), while the APs shifted toward the falling phase of the V_m_ oscillation for the opposite direction (*U* = 18,591.0, *p* = 0.00011; Fig. 5*B*). The APs phase angles were not shifted for the orthogonal direction (*U* = 9143.0, *p* = 0.85; Fig. 5*C*).

### Synaptic strengthening of L5 projections after the visual experience

**Figure 6.**
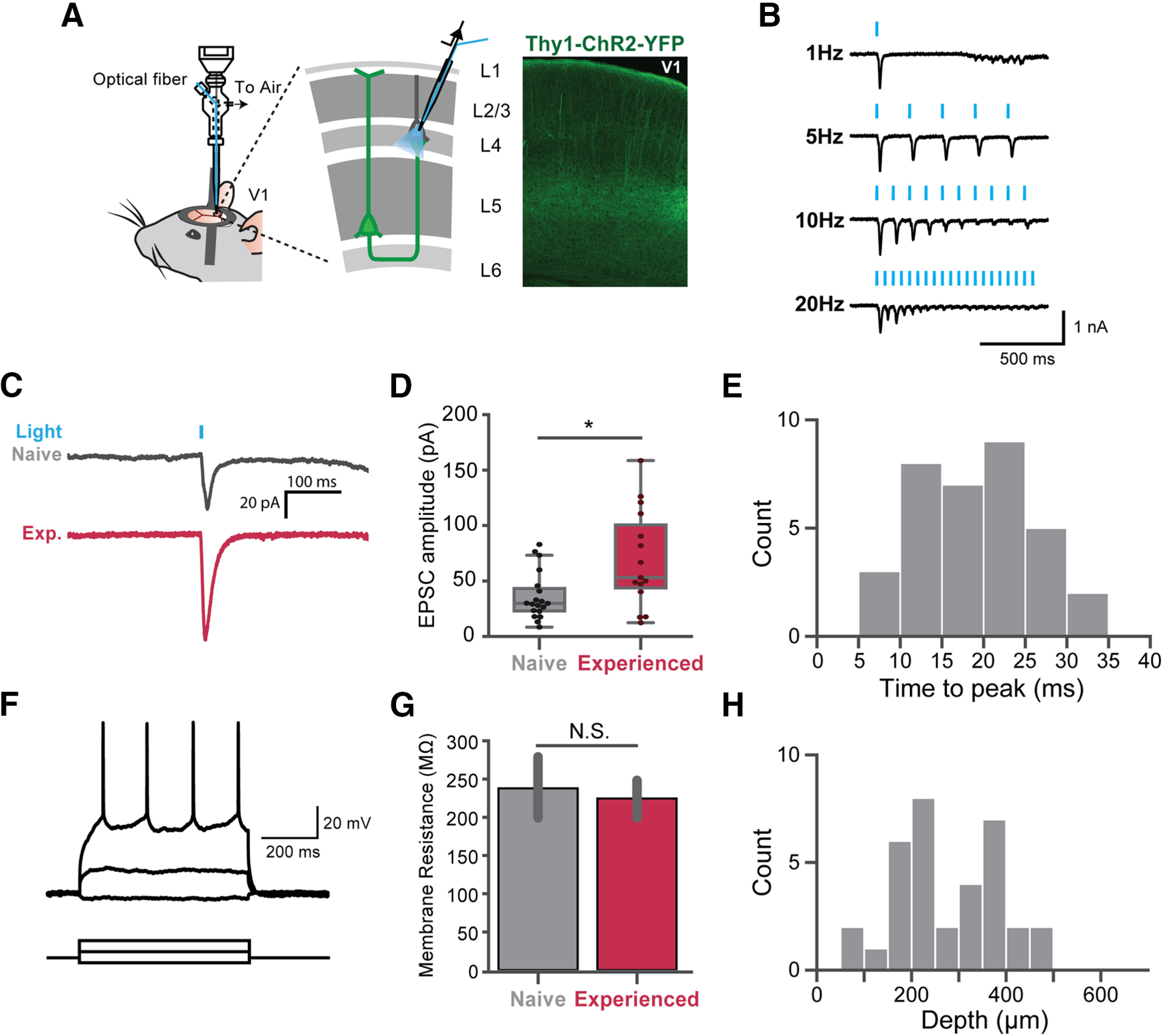
Synaptic strengthening of L5 projections by visual experience. ***A***, left, Recording schematic. Whole-cell recordings were made on random pyramidal cells in L2/3 and L4. Right, A V1 brain slice of Thy1-ChR2-YFP transgenic mice. ChR2 was sparsely expressed in V1 in these mice. Intracortical and intercortical projections from L5 ChR2-expressing cells were activated with 473-nm laser pulses. Light-induced EPSCs were recorded in voltage-clamp mode. ***B***, Example of 1-, 5-, 10-, and 20-Hz light pulses inducing presynaptic short-term depression in a patched neuron. ***C***, Examples of light-induced EPSC traces from single cells in a naive (top, gray) and an experienced mouse (bottom, red). ***D***, Averaged EPSC amplitudes of cells in naive (gray, 19 cells) and experienced mice (crimson, 15 cells). The EPSC amplitudes in experienced mice was significantly higher than naive mice. ***E***, The average time interval from the stimulus onset to the peak of the EPSC of all cells (34 cells). ***F***, Representative traces of step current injections in current-clamp mode; 10-nA increments were used for step current injection. Three steps are shown here. ***G***, The membrane resistance of cells in naive and experienced mice showed no significant difference. ***H***, The depth distribution of the responsive cells; *p < 0.05; N.S., not significant. Error bars indicate mean ± SEM.

To dissect the synaptic plasticity mechanisms underlying the emergence of the 5-Hz oscillation and the enhanced selectivity, we searched for synaptic changes that were induced by visual experience. One possibility was that the synaptic strength of L5 projections, which carry feedback information from recurrent circuits in V1 and higher visual areas, was changed. To test this hypothesis, we measured postsynaptic responses *in vivo* with photoactivation of channelrhodopsin-2 (ChR2)-positive synaptic terminals formed by L5 neurons. We used Thy-1-ChR2-YFP mice, where ChR2 is sparsely expressed in L5 pyramidal cells in V1 (Arenkiel et al., 2007). To improve the success rate and stability of the patches, we anesthetized mice with ketamine/xylazine. To optogenetically activate the ChR2-positive presynaptic terminals formed onto the patched cells, we used a specialized pipette holder with a port for the optical fiber, optopatcher (Katz et al., 2013). Blue laser light (wavelength 473 nm) was delivered through the tip of the recording pipette so that the intensity of light used for each cell was the same regardless of the depth (Fig. 6*A*).

We recorded neurons in V1 at a depth of <500 μm (Fig. 6*H*). To avoid directly recording from ChR2-expressing neurons, we used a light pulse train (5-ms pulses, 1, 5, 10, and 20 Hz) to induce short-term plasticity to confirm that the patched neurons did not express ChR2 (Fig. 6*B*). We then recorded light-induced EPSCs (Fig. 6*C*). For each cell, the responses of 10 trials were averaged. 19 out of 22 cells recorded in naive mice had responses. The 19 cells were recorded from 11 mice; 15 out of 19 cells had responses in experienced mice. The 15 cells were recorded from nine mice. The average time interval between the stimulus onset and the EPSC peak is 18.78 ms (Fig. 6*E*). It is longer than the time interval between the stimulus onset and the peak of the direct ChR2 current (∼8 ms) in a previous study (Boyden et al., 2005). EPSC amplitudes were significantly higher in experienced mice compared with naive mice (Mann–Whitney test, *U* = 79.0, *p* = 0.0144; Fig. 6*D*). We also measured the membrane resistance of responsive neurons in naive and experienced mice, finding no significant differences between the two groups (*U* = 93.0, *p* = 0.85; Fig. 6*F*,*G*).

### Thalamocortical synapses are weakened by visual experience

**Figure 7.**
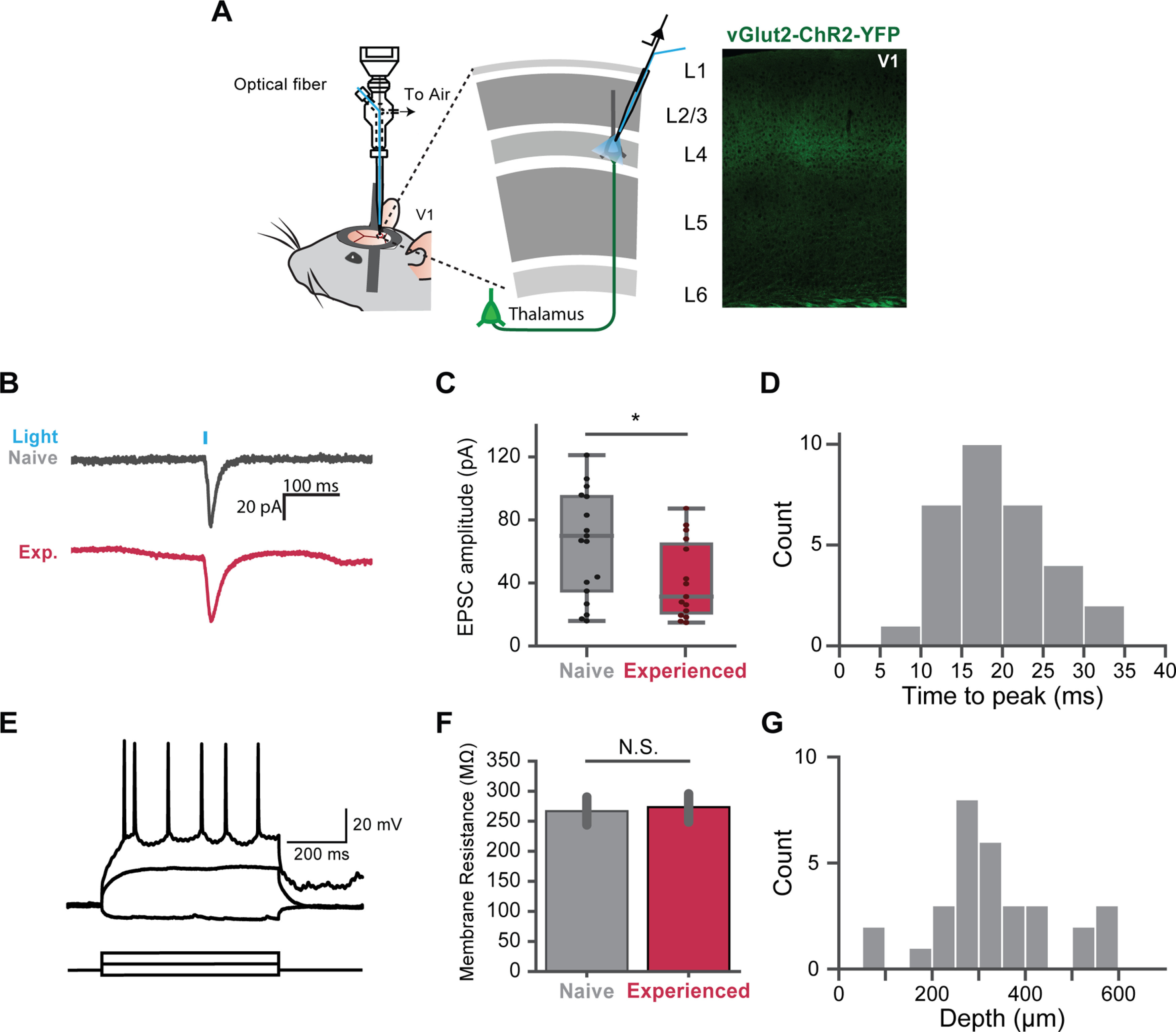
Thalamocortical synaptic strength is reduced by visual experience. ***A***, left, Whole-cell recordings were made on random neurons in L2/3 and L4. Right, V1 brain slice of VGlut2-ChR2-YFP transgenic mice. ChR2 was expressed in the thalamocortical projections. Light-induced EPSC were recorded in voltage clamp mode. ***B***, Examples of light-induced EPSC traces from single cells in a naive (top, gray) and experienced mouse (bottom, red). ***C***, Averaged EPSC amplitudes of cells in naive (gray, 16 cells) and experienced (crimson, 15 cells) mice. The EPSC amplitudes in experienced mice was lower than naive mice. ***D***, The average time interval from the stimulus onset to the peak of the EPSC of all cells (31 cells). ***E***, Representative traces of step current injections in current-clamp mode; 10-nA increments were used for the step current injection. Three traces are shown here. ***F***, Membrane resistance of cells in naive and experienced mice showed no significant difference. ***G***, The depth distribution of the responsive cells; *p < 0.05; N.S., not significant. Error bars indicate mean ± SEM.

We observed the decrease in the firing rate of visual responses, especially in the first peak of oscillation. This may result from decreased thalamocortical projection strength. To test whether the strength of thalamocortical projections was changed, we measured the postsynaptic responses *in vivo* with photoactivation of ChR2-positive thalamocortical terminals. To achieve the specific activation of thalamocortical projections, we used the VGlut2-ChR2-YFP mice, which express ChR2 specifically in the visual thalamus. In the VGluT2-ChR2 transgenic mouse line, ChR2 is expressed under the vesicular glutamate transporter 2 (VGluT2) promoter (Hägglund et al., 2010). VGluT2 has been shown to be specifically expressed in the thalamus but not in the V1 (Coleman et al., 2010). The axons which formed connections onto patched neurons were activated by 5 ms light pulses (Fig. 7*B*). EPSCs of 10 trials were recorded for each cell. A total of 17 out of 23 cells recorded in naive mice and 15 out of 26 cells recorded in experienced mice had monosynaptic responses (Fig. 7*C*). The average EPSC amplitudes were lower in experienced mice compared with naive mice (Mann–Whitney test, *U* = 64.0, *p* = 0.0141). We also measured the membrane resistance of these cells, but discovered no difference between the two groups (*U*= 72.0, *p* = 0.60; Fig. 7*F*).

### Network mechanism underlying 5-Hz oscillations following visual experience

**Figure 8.**
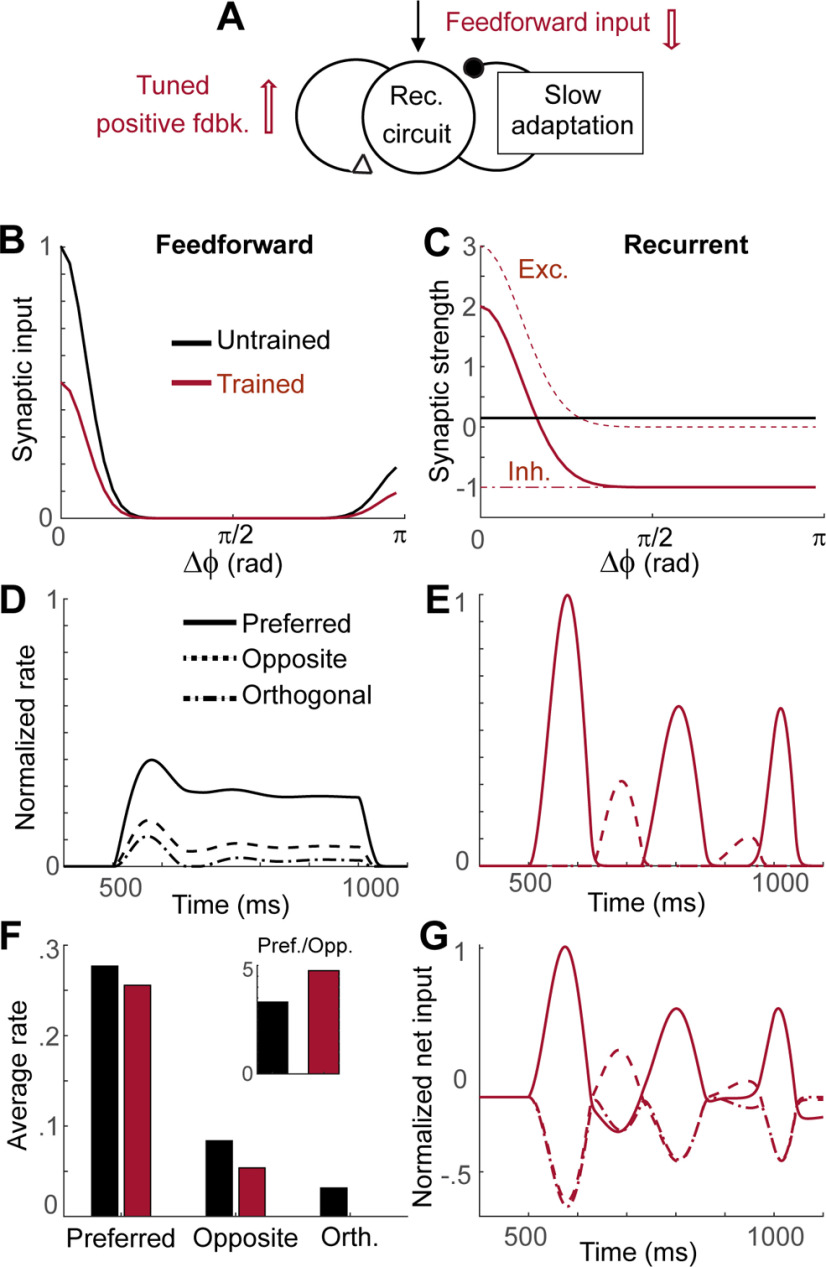
Network model with synaptic plasticity and slow adaptation reproduces the effects of visual experience. ***A–C***, Synaptic plasticity induced by learning. In the network with a ring structure and slow adaptation, feedforward and recurrent synapses undergo synaptic plasticity in the opposite directions (red arrows in ***A***), that is, a decrease of the feedforward inputs (***B***) and more structured and stronger connections of recurrent inputs (***C***). Here, Δφ represents difference between the preferred directions of neurons. ***D***, ***E***, Network activities before (***D***) and after learning (***E***). The stimulus is presented between 500 and 1000 ms, and all activities are normalized by the maximum firing rates after learning. Note the activity for orthogonal stimuli is zero because of thresholding. ***F***, Changes in average activities during the stimulus presentation with learning and the ratio of the activities in the preferred direction to that in the opposite direction (inset). ***G***, Net input normalized by its maximum after learning.

To obtain the mechanistic understanding of the effects of visual experience observed in V1, we considered a recurrent network model where activities of neurons are described by their firing rates (Fig. 8*A*). Previously, interactions between synaptic plasticity of recurrent connections and slow negative feedback such as spike frequency adaptation were proposed to account for similar effects of the visual experience observed in monkey inferotemporal cortex (Lim, 2019). We modified this model so that the network is organized in a one-dimensional ring structure where each unit represents a population of neurons having similar directional selectivity (Ben-Yishai et al., 1995; Ermentrout, 1998). We assumed that visual experience induces changes in both recurrent and feedforward inputs, as observed experimentally (Figs. 6, 7). Before learning, the network receives uniform recurrent input and asymmetric feedforward inputs with strong input at the stimulus direction and relatively weak but non-zero input in the opposite direction (Fig. 8*B*,*C*, black). The latter is a simplification of the experimental observation that thalamic inputs provide a primordial bias for directional selectivity in V1 (Lien and Scanziani, 2018). After learning, the feedforward inputs scale down uniformly, while recurrent inputs become structured with local excitation and global inhibition (Fig. 8*B*,*C*, red). Such changes in recurrent connections were inspired by the experimental observation of the reorganization of recurrent connections during the development (Ko et al., 2011) and biologically realistic models suggesting direction-based connectivity rules in recurrent synaptic strengths (Billeh et al., 2020). Note that we assumed that the emergence of oscillation after learning is intrinsic to V1 circuits with no oscillation in the feedforward or feedback inputs from other areas, similarly to the previous modeling works (Lien and Scanziani, 2018; Billeh et al., 2020).

The model reproduced the main effects of visual experience, enhanced directional selectivity, and low-frequency oscillations with a decrease in overall firing rates. Before learning, the network shows little oscillations but shows direction selectivity inherited by the feedforward input (Fig. 8*D*,*F*). After learning, strong low-frequency oscillation emerges in both preferred and opposite directions (Fig. 8*E*) because enhanced recurrent connections in the neurons with similar preferred directions lead to stronger positive feedback and its interaction with slow adaptation generates oscillation (Fig. 8*A*). The anti-phase oscillation in the opposite direction is led by net inhibitory inputs from neurons at the preferred directions, and the activity for the orthogonal directions is suppressed below the threshold (Fig. 8*E*). However, the net input shows the increased oscillation across all directions as an enhanced oscillation in voltage observed experimentally (Fig. 8*G*). Recurrent synaptic plasticity also leads to enhanced directional selectivity as the ratio of the average firing rate at the preferred direction to that of the opposite increased, while overall firing rates decrease because of depression in the feedforward inputs (Fig. 8*F*). Note that instead of uniform depression in the feedforward inputs, more structured changes of them can also contribute to the enhanced directional selectivity, yet feedforward synaptic plasticity alone is not sufficient to reproduce changes in response dynamics. Thus, both recurrent and feedforward synaptic plasticity is required, as observed experimentally.

## Discussion

Here, we demonstrated that a familiar visual stimulus could reliably evoke a 5-Hz oscillation in V1 of single cells in awake mice, confirming previous studies (Einstein et al., 2017; Kissinger et al., 2018). The oscillations were observed as both oscillatory fluctuations in V_m_ and firing rate and were accompanied by reduced firing at the visual stimulus onset. They were also strongly time-locked to the visual stimuli. They closely followed the stimuli and were highly coherent across trials. A similar observation was made in a previous study, where spontaneous 3- to 5-Hz V_m_ oscillations in V1 had a suppressive effect on visual responses (Einstein et al., 2017). We found that the oscillations were also not specific to the direction of the visual stimulus used for familiarization but could be recruited by all directions of visual stimuli in experienced mice. This observation confirmed a previous finding that the oscillation was more sensitive to the spatial frequency content rather than the direction of stimuli (Kissinger et al., 2018).

V1 has long been known to have specialized cell assemblies that extract specific features from visual stimuli (Hubel and Wiesel, 1959, 1962). The selectivity of these neurons can be changed not only in the developing brain but also in adults during learning. Previous studies have demonstrated that the selectivity of V1 can be modulated by perceptual learning (Schoups et al., 2001), classical conditioning (Goltstein et al., 2013), and reinforcement learning (Poort et al., 2015). In our study, we observed improved direction selectivity following the visual experience, which was accompanied by increased 5-Hz oscillation. The 4- to 7-Hz power of V_m_ was increased, and the firing rates of the visual responses decreased in experienced mice regardless of the direction of the stimuli. Although this reduction was not direction specific, the responses to the opposite and orthogonal directions demonstrated a larger proportion decrease than the preferred direction. Such changes led to the increased DS and OS. On the other hand, the selectivity of V1 neurons was improved while the neurons fired fewer APs, implying the encoding of visual stimuli in V1 could become more efficient. A very similar scenario of enhanced selectivity of V1 neurons had been demonstrated by non-specific activation of interneurons with optogenetics (Lee et al., 2012). The changes in visual responses and selectivity in this study were very similar to what we observed here after visual experience. The enhanced selectivity we observed could partially result from the experience-dependent recruitment of interneurons.

Reinforcement learning with a visual cue has been demonstrated to induce experience-dependent plasticity in adult V1, where the timing of the expected reward was reported by neural activity (Shuler and Bear, 2006; Chubykin et al., 2013). Noticeably, neural oscillations at a similar frequency range in V1 were also observed in rats undergoing reward training, and their duration reported the timing of the reward (Zold and Hussain Shuler, 2015; Levy et al., 2017). In our study, we also sought to find the synaptic strength changes associated with the 5-Hz oscillation. With optogenetically evoked EPSC measurements, we found that the L5 to superficial layers projection strength was increased, while the thalamocortical projection strength was decreased. The decreased thalamocortical projection strength could at least partially explain the reduction in firing rates at the onset of visual stimulation. These changes in synaptic strength may, at least in part, promote the emergence of oscillation and allow for enhanced selectivity. The synaptic changes we discovered were relatively large. We cannot determine whether such changes were uniform or were specific to the certain features of the visual stimulus from our results. However, since 5-Hz oscillations were found to be entrained by a wide range of visual stimuli yet were specific to the spatial frequency of the stimulus (Kissinger et al., 2018), the synaptic changes induced by experience could be structured with feature specificities. Meanwhile, the stimulus we used for the visual experience was also fairly strong compared with the natural visual input mice experience in their home cage. Our stimuli were 100% contrast, with a high number of repeats presented within a relatively short time period. These factors could contribute to the relatively large synaptic changes. Synaptic changes induced by the natural visual experience need to be further explored in the future.

Previous studies have suggested the brain state is correlated with the low-frequency activities in cortical areas (Harris and Thiele, 2011). Low-frequency V_m_ fluctuation of V1 neurons was stronger during the quiet wakefulness than the highly aroused state and was strongly correlated with pupil size (Reimer et al., 2014). Such low-frequency activities are mostly spontaneous and not temporally associated with visual stimuli. The increased low-frequency activity during quiet wakefulness was also accompanied by decreased high-frequency power in the γ range (Niell and Stryker, 2010). However, in our study, the 5-Hz oscillation was robustly evoked and strongly time-locked to the visual stimuli. Contrary to the low-frequency oscillations that happened during quiet wakefulness, the 5-Hz oscillation induced by visual experience was accompanied by increased γ range oscillation (Kissinger et al., 2018, 2020). Furthermore, although the magnitude of the visually evoked 5-Hz oscillation was higher in immobile mice, the oscillations were also strong during the mobile periods (Kissinger et al., 2020). Additionally, 3- to 5-Hz V_m_ oscillation was also found to happen in both high and low arousal states with similar likelihoods (Einstein et al., 2017). Thus, whereas the 5-Hz oscillations may be modified by the brain state, they are primarily triggered by the familiar stimulus.

Interestingly, the improvement of selectivity to familiar visual stimuli was also found in the primate inferior temporal cortex (ITC; Kobatake et al., 1998; Freedman et al., 2006). Similar to what we report here, the improvement of selectivity of ITC neurons was also achieved by temporally sharpened visual responses and decreased average firing rates (Freedman et al., 2006; Woloszyn and Sheinberg, 2012; Lim et al., 2015). Bursts of firing in a similar frequency range were also reported in ITC in response to the familiar stimuli. For familiar stimuli, three peaks of clustered APs firing were found in ITC neurons, while novel stimuli elicited only one peak (Freedman et al., 2006). Alternating familiar stimuli could also evoke periodic responses of ITC neurons, while novel stimuli did not (Meyer et al., 2014).

In our study, the V_m_ 8- to 12-Hz band demonstrated a trend toward an increase in magnitude following visual experience, which did not reach significance. However, the 8- to 12-Hz band ITPC was significantly increased for the orthogonal direction stimulus in experienced mice. Previous studies also observed elevated 8- to 12-Hz band power coincident with the rise of 4- to 8-Hz power in LFP recordings (Kissinger et al., 2018). Unlike the 5-Hz oscillation, the 8- to 12-Hz band likely only synchronizes a smaller ensemble of cells rather than the whole local cortical network. Otherwise, one would expect a similar elevation of 8- to 12-Hz ITPC in response to all directions rather than only the orthogonal direction. Although the 8- to 12-Hz band activity had been shown to convey feedback processing, especially in primates (van Kerkoerle et al., 2014; Michalareas et al., 2016), the significance of the increased 8- to 12-Hz band we observed remains to be explored in the future studies.

Neural oscillations have been proposed to provide well-controlled V_m_ fluctuations and time windows for synaptic plasticity in V1. The bidirectional outcome can strengthen the inputs that arrive at the peak of the oscillation and weaken the inputs that are out of phase (Buzsáki, 2002; Buzsáki and Draguhn, 2004). In our study, we found that the APs shifted toward the rising phase of the oscillation when the stimulus of preferred direction was presented. When the stimulus of the opposite direction was presented, the APs shifted toward the falling phase of the oscillation. The high ITPC in the experienced mice also showed strong phase-locking of single cell V_m_. These changes in temporally organized firing activity could promote spike-time dependent plasticity in V1, contributing to further improvements in orientation and direction selectivities and the reduction of randomness while potentially increasing the encoding precision. More research is required in the future to explore this possibility.

Recent modeling studies basing on primate ITC responses to novel and familiar visual stimuli agree with our findings (Lim et al., 2015; Lim, 2019). Based on the similar effects of visual learning observed in monkey ITC, different roles of synaptic plasticity at the feedforward and recurrent connections have been proposed. The feedforward and recurrent synaptic plasticity together enabled more efficient coding of the stimuli after learning as depression in the feedforward connections led to a decrease in overall activity, while Hebbian-type recurrent synaptic plasticity led to enhanced stimulus selectivity. On the other hand, potentiation in recurrent synapses was required so that stronger positive feedback through potentiation interacts with slow negative feedback such as adaptation and shapes the response dynamics. We modified this model according to the features of a V1 network and used it to reproduce our experimental data. One notable difference from the previous work was an overall increase of low-frequency oscillations across all directions in V1, while the strength of oscillations after learning depends on the preference to the object stimuli in ITC. This difference may arise from the properties of the stimuli and corresponding network architecture such that the response to the preferred and opposite stimuli was correlated through a ring-like network architecture compared with the response to two different object stimuli, and thus, the oscillations were spread across all directional stimuli.

The 5-Hz oscillations we observed in V1 extended for ∼500 ms to 1 s after the stimulus offset. Although the sustained oscillation could be explained by hysteresis in the recurrent dynamics of the local circuits, it is likely other mechanisms are involved as well. The rebound spiking activity caused by hyperpolarization-activated cyclic nucleotide-gated (HCN) channels could contribute to the excitation required for the oscillation. The feedback connections from higher cortical areas could also be involved in this process. Also, although we assumed that thalamic inputs are not oscillatory, previous experimental works described low-frequency dependent top-down modulation through thalamocortical loops, in particular, in tasks requiring more attention (Fiebelkorn et al., 2019). These possibilities could be explored in future experiments and addressed in a future extension of the model.
